# Carotid wall imaging with 3D_T2_FFE: sequence parameter optimization and comparison with 3D_T2_SPACE

**DOI:** 10.1038/s41598-021-81309-1

**Published:** 2021-01-26

**Authors:** Ang Yang, Xue Hong Xiao, Zhi Long Wang, Yong Xin Zhang, Ke Yi Wang

**Affiliations:** MR Department of Affiliated Zhongshan Hospital of Sun Yat-sen University, Sun Wendong Road No. 2, Zhongshan, Guangdong China

**Keywords:** Vascular diseases, Magnetic resonance imaging

## Abstract

Similar to sampling perfection with application-optimized contrast using different flip angle evolutions (SPACE), T2-weighted fast field echo (FFE) also has a black blood effect and a high imaging efficiency. The purpose of this study was to optimize 3D_T2_FFE and compare it with 3D_T2_SPACE for carotid imaging. The scanning parameter of 3D_T2_FFE was optimized for the imaging of the carotid wall. Twenty healthy volunteers and 10 patients with carotid plaque underwent cervical 3D_T2_FFE and 3D_T2_SPACE examinations. The signal-to-noise ratios of the carotid wall (SNR_wall_) and lumen (SNR_lumen_), and the contrast-to-noise ratios between the wall and lumen (CNR_wall_lumen_) were compared. The incidence of the residual flow signal at the carotid bifurcation and the grades of flow voids in the cerebellopontine angle region in the two sequences were also compared. The reproducibility of the two sequences was tested. No significant difference was observed between the two sequences in terms of the SNR_wall_ of healthy individuals and patients (P = 0.132 and 0.102, respectively). The SNR_lumen_ in the 3D_T2_FFE images was lower than that in the 3D_T2_SPACE images. No significant difference was observed between the two sequences in terms of the CNR_wall-lumen_. The incidence of the residual flow signal at the carotid bifurcation in 3D_T2_FFE was significantly lower than that in 3D_T2_SPACE. The grades of flow suppression in the cerebellopontine angle region in 3D_T2_SPACE was lower than that in 3D_T2_FFE. Both sequences showed excellent inter-and intra-observer reproducibility. Compared to 3D_T2_SPACE, 3D_T2_FFE showed stronger flow suppression while maintaining good imaging quality, which can be used as an alternative tool for carotid imaging.

## Introduction

Since the turbo spin echo (TSE) imaging technique was introduced in 1986^1^, it has become an indispensable tool in magnetic resonance imaging (MRI). The data acquisition speed of TSE is faster than that of ordinary spin echo (SE); however, for three-dimensional imaging, the scanning time may still be as long as dozens of minutes, which is a disadvantage in clinical settings. In the year 2000, SPACE technology was introduced^2,3^, which greatly accelerated data acquisition. Moreover, owing to its inherent flow sensitivity characteristics^4^, it has been used in some applications that require black blood, such as vessel walls^5,6^.

T2-weighted fast field echo (T2_FFE) is a steady-state gradient echo sequence^7^. In steady-state gradient echo sequences, once a steady state is reached, two types of signals are formed. The first signal is free induction decay (FID), which is formed after excitation with the most recent radio frequency (RF) pulse. The second component is SE, which is formed when the residual echo from the previous RF excitation is refocused by the current RF pulse^8^. Compared with other spoiler or refocused gradient echo sequences, T2_FFE only collects the spin echo (SE) component in the steady-state sequence, and the decay of the transverse magnetization is controlled by T2, which also significantly reduces the T2* effect. Due to the unbalanced spoiler gradient (intrinsic diffusion effect), T2_FFE is also sensitive to motion and flow^7,9,10^. In the past, T2_FFE was associated with a relatively low signal-to-noise ratio (SNR), which limited its clinical use^7^. With the growing popularity of high-field MR systems, T2_FFE is increasingly being used in clinical settings, such as in peripheral nerve imaging^11–13^. Meanwhile, T2_FFE employs gradient echoes for faster data acquisition, exhibits high imaging efficiency, and can obtain T2-weighted 3D isotropic images within a short time. Therefore, the purpose of this study was to optimize the 3D_T2_FFE and to compare it with 3D_T2_SPACE for MR imaging of the carotid artery wall at 3 T MR.

## Materials and methods

### Participants

This study was approved by the Institute Review Board of Affiliated Zhongshan Hospital of Sun Yat-sen University. We confirmed that all methods were performed in accordance with relevant guidelines and regulations (Declaration of Helsinki). Written informed consent was obtained from all the subjects prior to their enrollment. Thirty subjects (22 males and 8 females) were enrolled in this study, including twenty healthy volunteers (mean age: 47.2 ± 13.3 years) and 10 symptomatic patients (mean age, 57.5 ± 9.2 years,) with known carotid plaque disease (inclusion criteria: > 50% stenosis caused by plaque; exclusion criteria: contraindications for MR imaging, severe motion artifacts).

### MRI protocols

All the subjects underwent MRI with a 3.0 T MR scanner (Achieva TX Systems, Philips, Netherlands) equipped with a Sense_NV_16 coil (Head and neck coil with 16 channels). Before the examination, the subjects were reminded to avoid swallowing and neck movement. The MRI examinations were performed with three-dimensional isotropic coronal acquisition, and the scanning range included the common carotid and internal carotid arteries, cerebellopontine angle cistern upward, and the superior margin of sternoclavicular joint downward.

**Figure 1:**
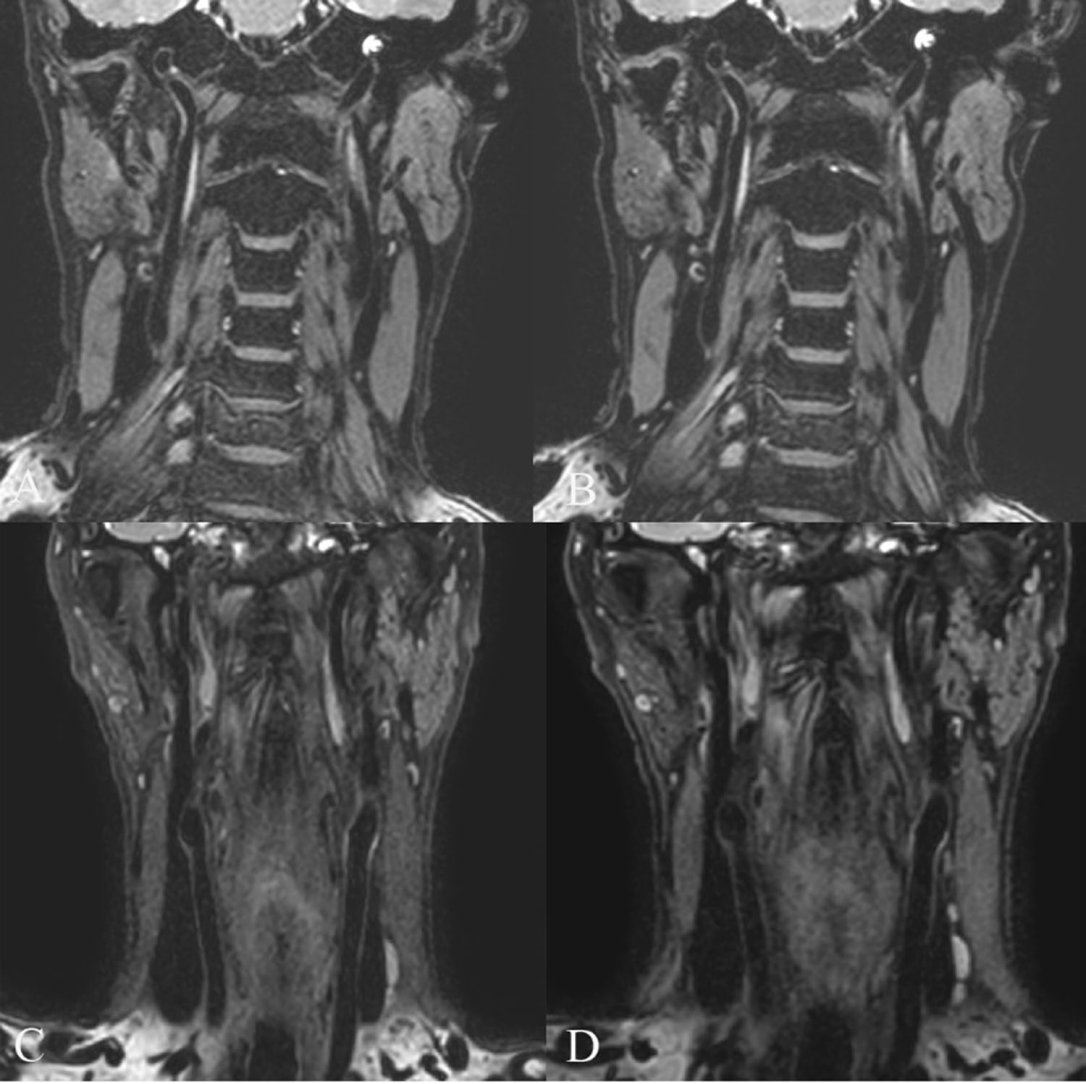
(**A**) Imaging with water-fat shift 0.5, TR 7 ms, TE 3.3, flip angle 25; (**B**) imaging with water-fat shift 0.8, TR 8 ms, TE 3.9, flip angle 25, other parameters are the same as those in (**A**). Compared with (**A**), (**B**) has less noise and higher image quality; (**C**) imaging with flip angle 25, TR 8 ms, TE 3.9; (**D**) imaging with flip angle 35, TR 8 ms, TE 3.9, other parameters are the same as those in (**C**). It can be found that although the T2_weighting of (**D**) is stronger (note that the signal of lymph nodes is higher), but the SNR of (**D**) is reduced (note that the display of the common carotid artery wall is relatively poor).

First, the carotid artery wall imaging pre-experiment with 3D_T2_FFE was performed on nine healthy volunteers (these nine healthy volunteers were not among the 30 subjects in the actual study). The imaging parameters were optimized based on the characteristics of T2_FFE. The repetition time (TR) was set to 8 ms, which was a balance point for obtaining a good SNR and reducing bulk motion artifact. TE was set to the shortest to reduce the sensitivity to cardiac motion and respiratory motion. The water-fat shift was set at 0.8 to make TE close to half of the TR (when TE was set to the shortest), which can reduce the dephasing in T2_FFE. The flip angle was set at 25° to obtain a good T2 contrast (Fig. 1). The principle of the selective excitation technique (PROSET) was chosen as the fat-suppression method to improve the contrast between the wall and the surrounding fat.

Twenty volunteers and 10 patients were scanned with both 3D_T2_FFE (with optimized imaging parameters) and 3D_T2_SPACE, while the scanning of 3D_T2_SPACE, the voxel resolution, field of view, slices, slice orientation and sense were set to be the same as 3D_T2_FFE, the TSE factor was set to 100 in order to gain a substantial black blood effect (Table 1). The acquisition times of 3D_T2_FFE and 3D_T2_SPACE were approximately the same.

**Table 1:** Parameters of the 2 MRI sequences.

	3D_T2_FFE	3D_T2_SPACE^a^
Field of view, mm	250 × 190 × 48	250 × 190 × 48
ACQ voxel, mm	1.2 × 1.2 × 1.2	1.2 × 1.2 × 1.2
REC voxel, mm	0.6 × 0.6 × 0.6	0.6 × 0.6 × 0.6
Slices	80	80
Fold over suppression	Yes	Yes
Slice orientation	Coronal	Coronal
Sense	2.3 (RL)	2.3 (RL)
Scan mode	3D	3D
Technique	FFE	SE
Fast mode	None	TSE
Shot mode	–	Multishot
Profile order	–	Linear
TSE factor	–	100
Start-up echoes	–	6
Flip angle, degree	25	90
TE, ms	3.9 (shortest)	172^b^
TR, ms	8	2000
Water-fat shift	0.8	Maximum
Fat suppression	Proset	SPIR
Shim	Default	Default
NSA	4	1
Scan time, min	2:20	2:10

FFE, fast field echo; NSA, number of signals averaged; SE, spin echo; SPIR, spectral presaturation inversion recovery; TSE, turbo spin echo; Proset, principle of selective excitation technique; ACQ, acquisition; REC, reconstruction; RL, right to left direction.

^a^3D view, MSK T2 FS.

^b^TEequiv, 70 ms.

### Image analysis

**Figure 2:**
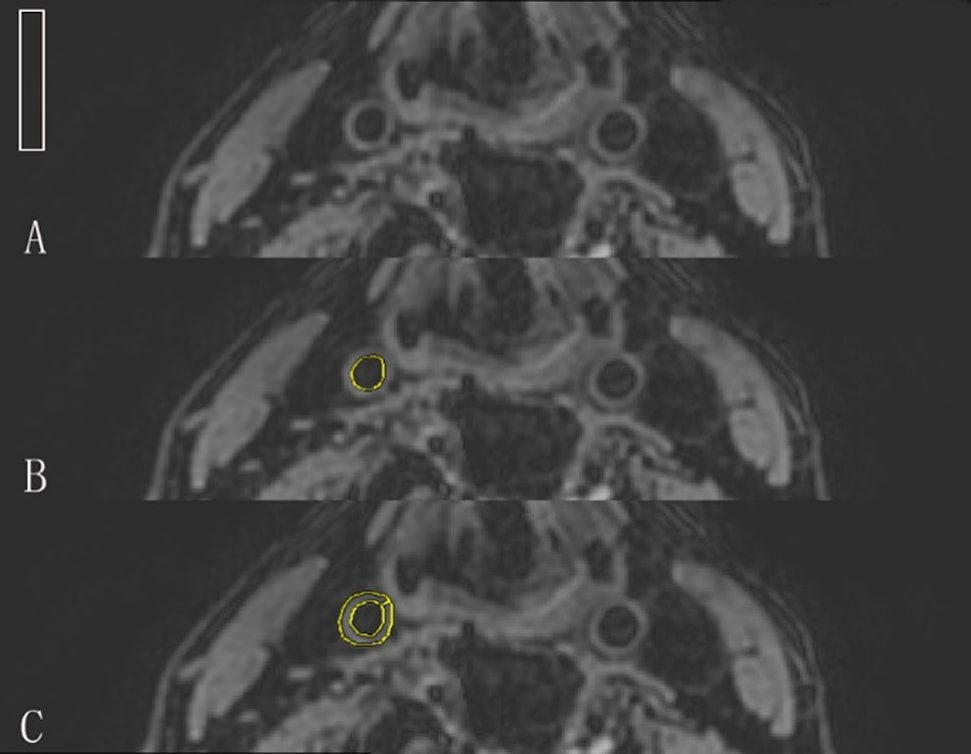
(**A**) One white region of interest (ROI) obtained from an artifact free background for the noise estimation. This ROI was positioned on the same layer as ROI of carotid wall and lumen and was of the same relative size on different subjects; (**B**) one yellow ROI obtained from the lumen of the common carotid artery (CCA); and (**C**) one yellow ROI obtained from wall of the CCA.

All the data were imported into clinical workstations (ONIS 2.0 free version, Tokyo, Japan, http://www.onis-viewer.com/) for data analysis. The images were quantitatively evaluated by two experienced radiologists (with 5 and 7 years of experience, respectively) with a randomized and blinded reading method. Data were acquired as the average of each radiologist’s measurements. SNR was calculated according to the equation SNR = SI/(1.53 × SD_Bg_), where SI is the signal intensity and SD_Bg_ is the standard deviation of the noise determined in an artifact-free background region. The factor 1.53 is the Rayleigh noise distribution correction factor^14^. The carotid wall signal intensity was measured as the mean signal intensity within a region of interest drawn on the vessel wall of the distal common carotid artery (CCA). The luminal signal intensity was calculated as the mean signal intensity within regions of interest drawn to contain the carotid arterial lumen at the same layer. The contrast-to-noise ratio of the wall to the lumen (CNR_wall_lumen_) was determined using the equation CNR_wall_lumen_ = (SNR_wall_ − SNR_lumen_). For the volunteers, the right CCA was measured, and for the patients, the CCA with the most obvious plaque was measured (Fig. 2). To test the reproducibility of the two sequences, inter- and intra-observer studies were performed. The SNR of the CCA measured by the two radiologists was used for the inter-observer analysis. The radiologist with 5 years’ experience measured the SNR of the CCA again 2 months later, and his first and second measurements were used for the intra-observer analysis.

**Figure 3:**
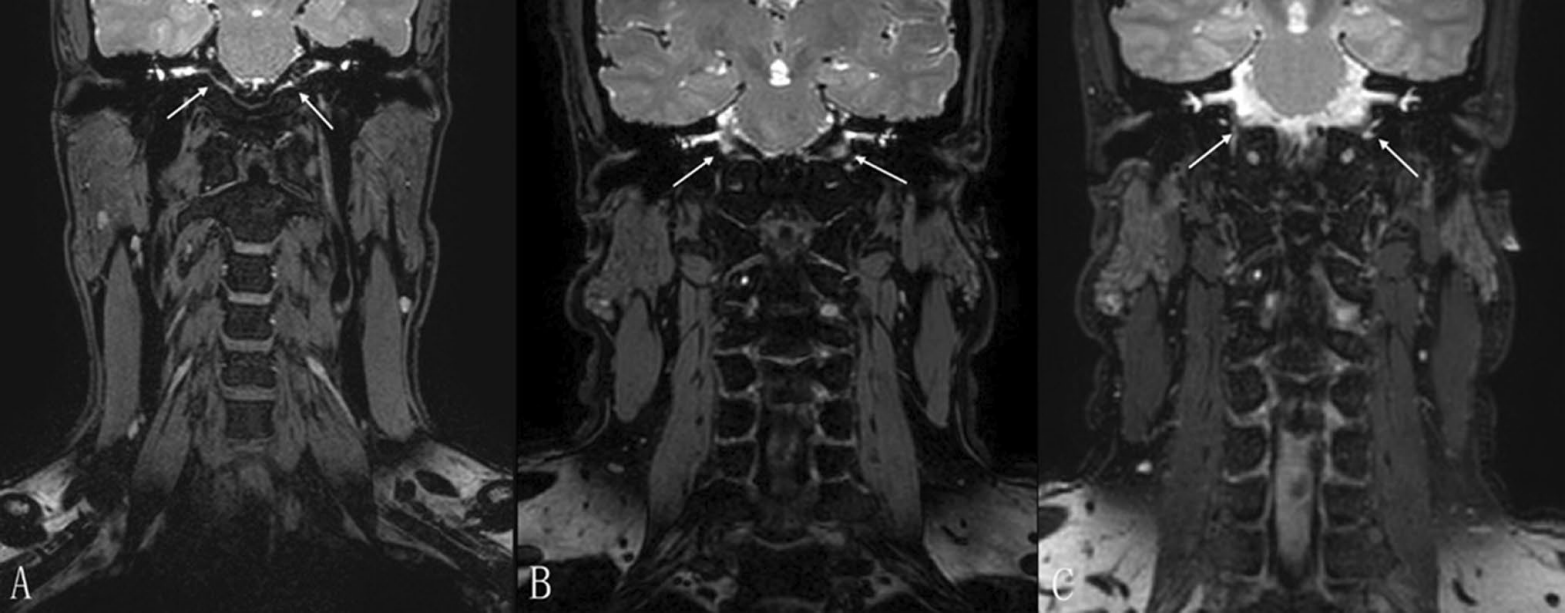
Examples of different grading scales of flow suppression in the cerebellopontine angle region (arrow). (**A**) Most flow suppression (grade 3) presented on 3D T2-weighted fast field echo (3D_T2_FFE). (**B**) Partial flow suppression (grade 2) presented on 3D_T2_FFE. (**C**) No flow suppression (grade 1) presented on 3D_T2_SPACE.

For the qualitative analysis, all the images were retrospectively evaluated by two experienced radiologists (with 6 and 10 years of experience, respectively) with a randomized and blinded reading method. MR images were examined in a subjective manner, and the final evaluation was agreed upon by consensus of the two radiologists. A 3-point grading scale (3 = most flow suppression; 2 = partial flow suppression; 1 = no flow suppression) was used to evaluate the flow sensitivity in the cerebellopontine angle region (Fig. 3). The presence or absence of a residual flow signal at the carotid bifurcation was also recorded for each sequence.

### Statistical analysis

Statistical analyses were performed using SPSS software (v.19.0). The threshold for statistical significance was set at P < 0.05. The SNRs of the two MRI sequences were compared using the paired-samples *t*-test (*two*-*tailed)*. “Crosstab” Chi-squared analysis was used to analyze the presence of a residual flow signal at the carotid bifurcation in the two sequences. The non-parametric two-related samples test was used to compare the degree of flow suppression in the cerebellopontine angle region. The inter- and intra-observer reproducibility was evaluated using the intraclass correlation coefficient (ICC). ICC < 0.4 indicates poor reproducibility, 0.4 < ICC < 0.75 indicates good reproducibility, and ICC > 0.75 indicates excellent reproducibility^15^.

## Results

**Figure 4:**
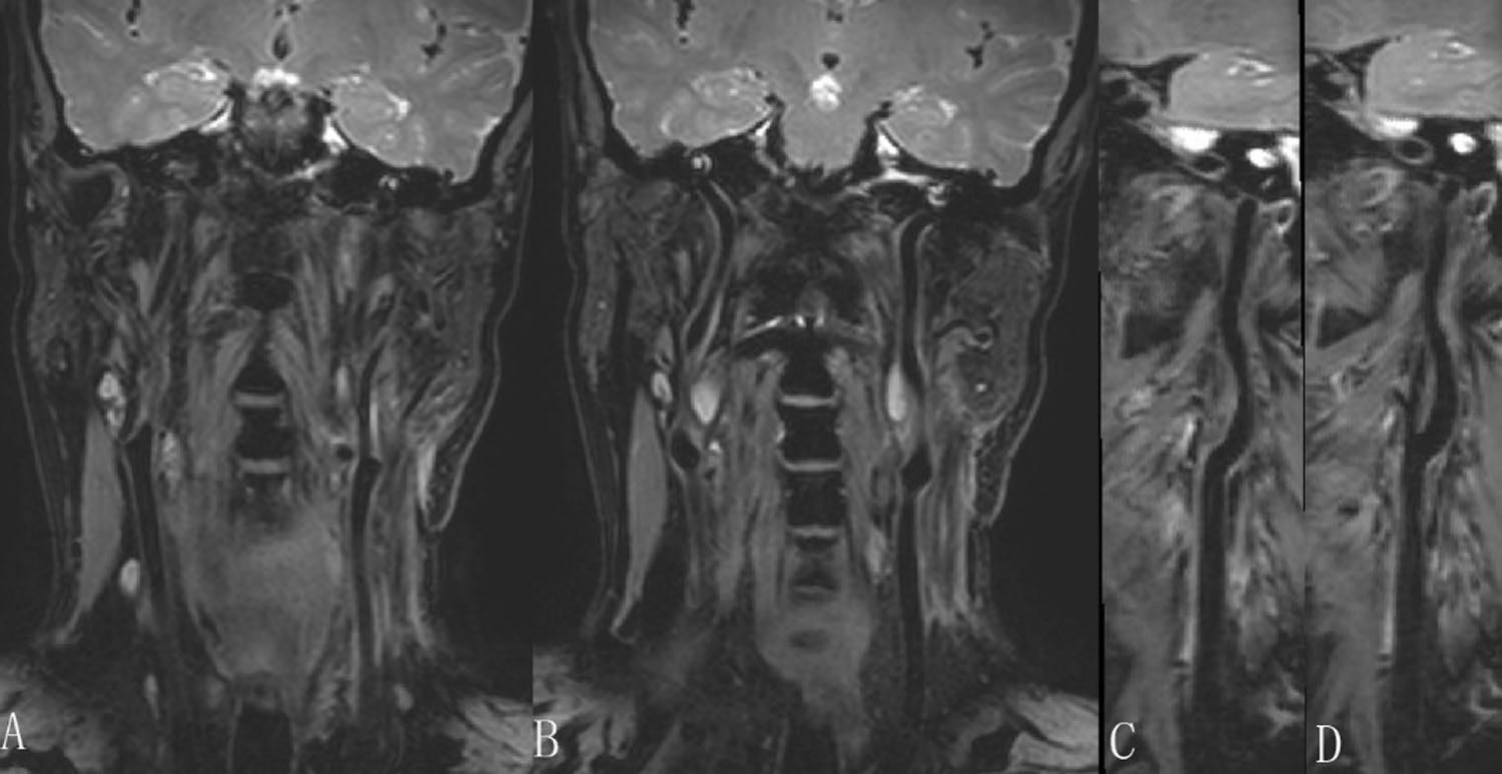
The carotid artery wall in the neck area were well displayed on 3D T2-weighted fast field echo. (**A**,**B**) coronal view, and (**C**,**D**) sagittal view.

The 3D_T2_FFE clearly displayed the CCA and internal carotid wall (Fig. 4). The SNR_wall_ in the 3D_T2_FFE images was comparable to that in the 3D_T2_SPACE images (P = 0.132 and 0.102 for the volunteer and patient groups, respectively). On the contrary, the SNR_lumen_ in the 3D_T2_FFE images was lower than that in the 3D_T2_SPACE images (P = 0.029 and 0.035 for the volunteer and patient groups, respectively). No significant difference was observed between the two sequences with respect to the CNR_wall-lumen_ (P = 0.653, 0.222 for the volunteer and patient groups, respectively) (Table 2).

**Table 2:** Comparison of the SNR and CNR of different structures in the two MRI sequences.

	3D_T2_FFE vs. 3D_T2_SPACE		*P* value
**SNR**_**wall**_			
Volunteer	55.6 ± 12.4	59.0 ± 15.4	0.132
Patient	46.3 ± 12.6	57.1 ± 18.9	0.102
**SNR**_**lumen**_			
Volunteer	17.2 ± 5.2	21.4 ± 8.2	0.029*
Patient	14.1 ± 4.2	18.7 ± 7.3	0.035*
**CNR**_**wall-lumen**_			
Volunteer	38.4 ± 8.3	37.6 ± 11.2	0.653
Patient	32.2 ± 9.9	38.4 ± 12.7	0.222

SNR, signal-to-noise ratios; CNR, contrast-to-noise ratio; FFE, fast field echo; SPACE, sampling perfection with application-optimized contrast using different flip angle evolutions.

*P < 0.05.

**Figure 5:**
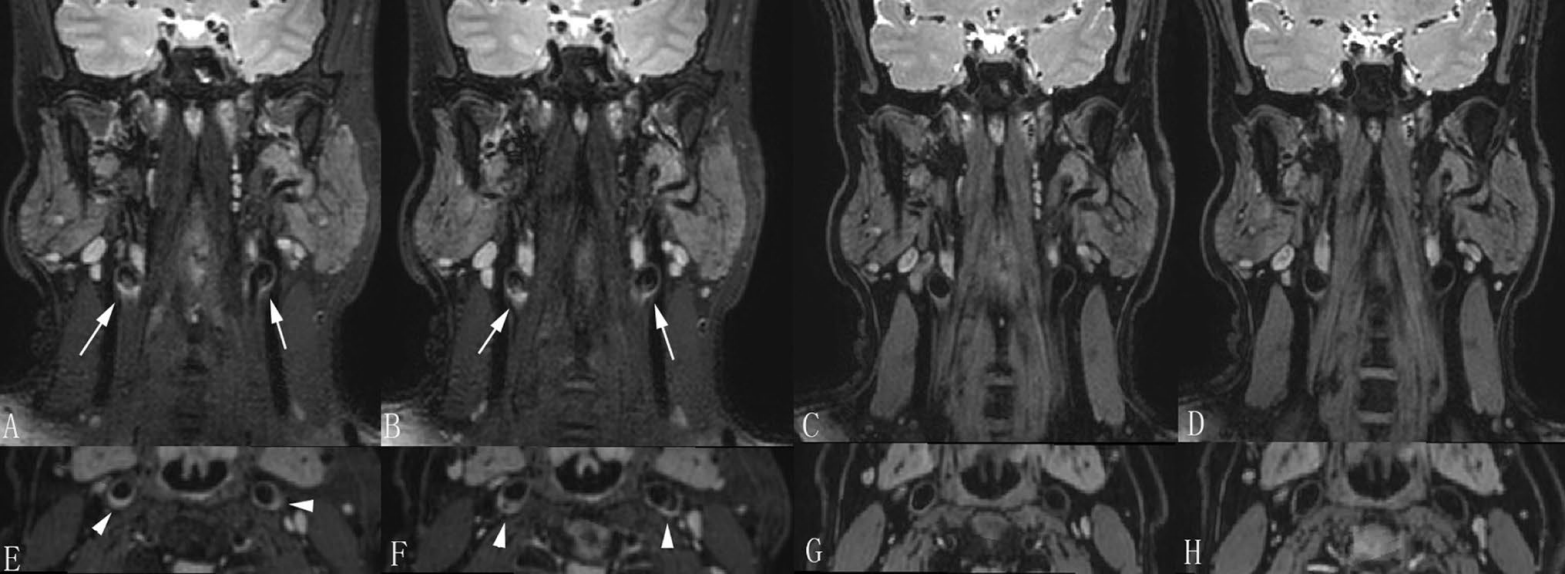
Examples of residual flow artifacts at the carotid bifurcation. (**A**,**B**,**E**,**F**) Presence of residual flow artifacts at the carotid bifurcation (arrows on the coronal plane and arrowheads on the reconstructed transverse plane) presented on 3D T2-weighted fast field echo (3D_T2_SPACE). Note that the artifacts may lead to overdiagnosis of plaques. (**C**,**D**,**G**,**H**) No residual flow artifacts at the carotid bifurcation were presented on 3D_T2_FFE (Same subject).

Four subjects presented with residual flow signals in 3D_T2_FFE (two volunteers and two patients), while 14 subjects presented with residual flow signals in 3D_T2_SPACE (nine volunteers and five patients) (Fig. 5). The incidence of the residual flow signal in 3D_T2_FFE was significantly lower than that in 3D_T2_SPACE (Table 3). In 28 subjects, the grades of flow suppression in the cerebellopontine angle region in 3D_T2_SPACE were lower than those in 3D_T2_FFE. The grades were equal in the two sequences only in two subjects. The grade of flow suppression in the cerebellopontine angle region in 3D_T2_SPACE was lower than that in 3D_T2_FFE (P < 0.001).

**Table 3:** Residual flow signal at the carotid bifurcation in the two MRI sequences.

Sequences	3D_T2_FFE	3D_T2_SPACE	P value
**Positive rate (each side of carotid bifurcation was counted)**			
Volunteer	3/40	11/40	0.017^a^,*
Patient	3/20	8/20	0.049^a^,*
P value	0.390^b^	0.326^b^	

MRI, magnetic resonance imaging; FFE, fast field echo; SPACE, sampling perfection with application-optimized contrast using different flip angle evolutions.

*P < 0.05.

^a^3D_T2_FFE vs. 3D_T2_SPACE.

^b^Volunteer vs. patient.

For measurements of CCA, both 3D_T2_FFE and 3D_T2_SPACE showed excellent intra-(ICC = 0.902, 0.921, respectively) and inter-observer (ICC = 0.833 and 0.858, respectively) reproducibility (Table 4).

**Table 4:** Intra- and inter-observer variability of the data analysis in the two MRI sequences.

	3D_T2_FFE			3D_T2_SPACE		
	ICC	95%CI	P value	ICC	95%CI	P value
Intra-observer	0.902	0.762–0.976	< 0.001*	0.921	0.797–0.968	< 0.001*
Inter-observer	0.833	0.575–0.913	< 0.001*	0.858	0.613–0.905	< 0.001*

95% CI, 95% confidence interval; FFE, fast field echo; SPACE, sampling perfection with application-optimized contrast using different flip angle evolutions; MRI, magnetic resonance imaging.

*P < 0.05.

## Discussion

We optimized the 3D_T2_FFE sequence by a pre-experiment in order to make it better for carotid wall imaging. We also compared the 3D_T2_FFE and 3D_T2_SPACE sequences for the MR imaging of the carotid wall. We found that the imaging quality of the carotid wall achieved with 3D_T2_FFE was comparable to that with 3D_T2_SPACE. In addition, the incidence of the residual flow signal at the carotid bifurcation in 3D_T2_FFE was significantly lower than that in 3D_T2_SPACE. High-grade flow suppression in the cerebellopontine angle region appeared more frequently on the 3D_T2_FFE sequence, which implies that 3D_T2_FFE has a more complete flow signal suppression than 3D_T2_SPACE.

In T2_FFE, while FID is destroyed by the crusher gradient on the slice-selection and read-out axes, a balance gradient (phase rewinder) is used in the phase-encoding axis to preserve the transverse magnetization^7,16^. Unlike FFE that employs both FID and SE signals, T2_FFE only makes use of the SE signal, and its effective TE is > TR. It is T2-weighted and has negligible T2* weighting. In addition, the features of T2_FFE using the SE component render it insensitive to local magnetic field inhomogeneity^9,11,17^. As a gradient-spoiled sequence, T2_FFE is particularly sensitive to diffusive motion, especially when the spoiler precedes imaging^8,18,19^. All these features render it suitable for black-blood vascular wall imaging.

In this study, we used a three-dimensional acquisition method. Steady-state gradient echo is a fast-imaging method with high acquisition efficiency. In combination with 3D Fourier coding, it can obtain high-resolution non-interpolated 3D images in a reasonably short time [four NSA (the number of signals averaged) only took a little more than 2 min]. The TR was set to 8 ms, which is a balance point for obtaining a good SNR, reducing its scanning time, and avoiding bulk subject motion (The longer the TR, the larger the SNR, larger motion artifact)^7^. More NSA, appropriate TR, and high field strength made that the SNR_wall_ on 3D_T2_FFE was comparable to that on 3D_T2_SPACE.

In this study, 3D_T2_FFE was combined with the PROSET fat suppression technology to improve the contrast between the wall and surrounding fat, which also effectively attenuates the chemical shift artifacts. Compared with spectral prestarvation with inversion recovery (SPIR), PROSET has the following advantages: (1) insensitivity to the B0 field, B1 field inhomogeneity, and ability to obtain a uniform fat suppression effect; (2) high SNR; and (3) inhibition of fat signal without affecting the steady state^20,21^. The contrast of T2_FFE is highly dependent on the flip angle; increasing the flip angle was shown to increase the transverse magnetization, which subsequently increased the SE signal, leading to enhanced T2-weighting^7,17^. Therefore, we use a relatively large flip angle (25°) to obtain heavier T2-weighting. As a steady-state gradient echo sequence, it is often desirable to minimize TE to reduce the sensitivity to cardiac motion and respiratory motion. Unlike the gradient-spoiled sequence, reduced dephasing (minimizes unwanted phase dispersions due to T2* processes) in T2_FFE should make TE close to half of TR (acquisition of signal as close as possible before the second pulse)^8,22^. We used a 0.8 water-fat shift to make the TE close to half of TR.

Conventional TSE is limited by the decay effect of T2, and the echo chain cannot be very long (typically < 30); otherwise, the ambiguity effect caused by T2 decay will be very serious. Coupled with the limits of the RF absorption rate, the data acquisition efficiency of conventional TSE cannot meet the needs of three-dimensional imaging. The emergence of SPACE solves the 3D TSE imaging problem^3^. SPACE has the following characteristics: (1) it initially uses a small flip angle, wherein the magnetization vector is mostly retained in the longitudinal direction; subsequently, a series of optimized flip angles are used to maintain a stable level of transverse magnetization after each pulse excitation. This helps avoid the blurring effect caused by the decay of the long echo chain. (2) Due to the fact that the refocusing pulse is no longer a uniform large angle, the specific absorption rate is also significantly reduced. Therefore, even on the 3.0 T system, the echo chain length can easily reach more than one hundred. (3) The design of the variable flip angle chain causes the transverse and longitudinal magnetization vectors to be converted to each other, such that the phase accumulated by the motion is also converted between the SE and the stimulated echo. When the SE and the stimulated echo are superimposed to form a signal, the phase accumulated by the motion attenuates the signal. Therefore, SPACE has a black-blood effect^4–6^. Among the imaging parameters of SPACE, the motion sensitivity is determined by the minimum flip angle. The smaller the minimum flip angle, the stronger the motion sensitivity of SPACE. The minimum flip angle of SPACE is indirectly controlled by the echo train length (ETL). When the ETL is larger, the minimum flip angle will be smaller^23^. In this study, the ETL of SPACE is 100, which is the balance point for adequate flow sensitivity and sufficient SNR. Nevertheless, we found that 3D_T2_FFE exhibited more complete flow suppression than 3D_T2_SPACE, as shown by the greater residual flow signal at the carotid bifurcation and higher SNR_lumen_ observed with the latter. This may be attributable to the fact that the unbalanced crusher gradient exacerbates the flow sensitivity of the 3D_T2_FFE images. In the past, motion sensitivity limited the clinical application of T2_FFE, but for this study, the motion sensitivity of 3D_T2_FFE obtained a better black blood effect without compromising its image quality, as it showed excellent inter- and intra-observer reproducibility.

Although 3D_T2_FFE has some advantages over 3D_T2_SPACE in displaying the vessel walls, it has some limitations in displaying the vessel wall in the cervical root region. This is due to the severe magnetic field inhomogeneity in this area; even PROSET could not achieve uniform fat suppression. Of course, SPIR used in 3D_T2_SPACE could not achieve uniform fat suppression in the cervical root region either. Although there is no significant difference between the SNR_wall_ of 3D_T2_FFE and that of 3D_T2_SPACE, the mean value of SNR_wall_ of 3D_T2_FFE is lower than that of 3D_T2_SPACE, especially in patients. This may be due to the fact that although T2 FFE collects spin echo, its T2_weighting is not as strong as TSE, and the SNR_wall_ is even higher when there are plaques.

With the further increase of field strength and the application of acceleration technologies such as sparse acquisition, the defects of T2_FFE will be overcome to a certain extent, and there should be more clinical application, such as small nerve, small vessel and other applications that require both black blood and T2 contrast.

## Conclusion

3D_T2_FFE has greater sensitivity to blood flow and can achieve a better black blood effect over 3D_T2_SPACE for displaying the carotid wall. Moreover, the SNR and CNR of 3D_T2_FFE are comparable to those observed with 3D_T2_SPACE in a 3 T system. It can be used as an alternative tool for carotid imaging.
